# Calibration of a Cohesive Model for Fracture in Low Cross-Linked Epoxy Resins

**DOI:** 10.3390/polym10121321

**Published:** 2018-11-28

**Authors:** Dery Torres, Shu Guo, Maria-Pilar Villar, Daniel Araujo, Rafael Estevez

**Affiliations:** 1 Departamento de Ciencia de los Materiales e Ingeniería Metalúrgica y Química Inorgánica, Campus de Puerto Real, 11510 Cádiz, Spain; Dery.Torres@uca.es (D.T.); Pilar.Villar@uca.es (M.-P.V.); Daniel.Araujo@uca.es (D.A.); 2 Unievrsité de Grenoble, CNRS, F-38000 Grenoble, France; shugeladi2004@hotmail.com

**Keywords:** crazing, cohesive zone, epoxy RTM, elastic-viscoplastic crack tip fields

## Abstract

Polymer-based composites are becoming widely used for structural applications, in particular in the aeronautic industry. The present investigation focuses on the mechanical integrity of an epoxy resin of which possible damage results in limitation or early stages of dramatic failure. Therefore, a coupled experimental and numerical investigation of failure in an epoxy resin thermoset is carried out that opens the route to an overall micromechanical analysis of thermoset-based composites. In the present case, failure is preceded by noticeable plasticity in the form of shear bands similar to observations in ductile glassy polymers. Thus, an elastic-visco-plastic constitutive law initially devoted to glassy polymer is adopted that captures the rate- dependent yield stress followed by softening and progressive hardening at continued deformation. A general rate-dependent cohesive model is used to describe the failure process. The parameters involved in the description are carefully identified and used in a finite element calculation to predict the material’s toughness for different configurations. Furthermore, the present work allows investigation of nucleation and crack growth in such resins. In particular, a minimum toughness can be derived from the model which is difficult to evaluate experimentally and allows accounting for the notch effect on the onset of failure. This is thought to help in designing polymer-based composites.

## 1. Introduction

Epoxy resins are used as a matrix in a large number of thermoset- based composites due to the large number of components that can react with the epoxy resin to form composites with a very wide range of properties [1]. However, it is necessary to gain insight in the fracture response and the mechanisms underlying failure of epoxy resin under various loading rates for reliable design in engineering applications.

The fracture mechanism in epoxy resins remains to be identified and at the moment there is no general agreement in the thermosets community about the physics underlying failure. In thermosets with a high cross-link density, Kinloch [2] considers that crazing as observed in glassy polymers is not likely to occur. The main argument for this point of view is that chain disentanglement or chain scission is not likely to take place in thermosets while this is key in the formation of the web of fibrils. However, some evidence of craze-like structures are reported in toughened or low cross-linked thermosets [3,4,5]. In this study, a rate-dependent cohesive model is adopted for the description of the fracture mechanism, which is general enough to capture the foregoing points of view. The bulk response is also rate-dependent as evidenced in uniaxial compression tests, with a rate-dependent yield stress followed by softening and progressive hardening. When bulk viscoplasticity takes place prior to failure, the material’s toughness is then governed by the competition between both rate- dependent mechanisms (see [6]). How much the toughness appears rate dependent, in the case of a low cross-link epoxy, is investigated here. The possible dependence will be used as an indicator where a mechanism similar to that found in glassy polymer takes place or not.

The goal of this study is also to predict the toughness of epoxy resins for blunt- and sharp-notched specimens. The material under consideration is used in the aeronautical industry usually combined with nanoparticles to improve its fracture resistance or with additional long carbon fibers. The present study is devoted to the analysis and description of a neat epoxy. The preparation of a sharp or natural crack as recommended by the ESIS-TC4 technical committee is difficult without inducing initial plasticity by tapping or sliding. This observation also holds for ductile solid polymers. On the contrary, machining a blunted notch without generating plastic deformation is tractable.

In this work, specimens with different blunted notch radii carefully machined are used for the fracture tests. The fracture characteristics (force at rupture, toughness) are used to identify the cohesive parameters for fracture in the epoxy under consideration and its validation from a comparison between the predictions and the toughness measurements for different notch radii and loading rates is presented. Based on this identification, a prediction of the toughness for a sample with a natural crack is derived. The methodology reported here could then be used to characterize and model the fracture of ductile polymers, thermosets, or thermoplastics.

The paper is organized as follows. The bulk constitutive law is presented first, which realistically describes the stress-strain response. The material’s parameter identification is performed with data derived from uniaxial compression tests at various strain rates.

A second part is devoted to the description of the cohesive model for fracture. The cohesive parameters are identified through an inverse method for a fracture test configuration with a given notch radius. A validation is performed by comparing the toughness predictions and measurements for other notch radii, under various loading rates. The toughness predicted for a sample with a natural crack is reported, and the rate dependence commented. A discussion on the possible mechanism underlying fracture in the thermosets under consideration is then reported. Finally, concluding remarks complete the presentation.

Tensor are denoted by bold-face symbols, · the scalar product, a ${\left(\right)}^{{}^{\prime }}$ identifies the deviatoric part of a second-order tensor and I is the identity second-order tensor.

## 2. Mechanical Response of the Epoxy Resin RTM: Experiments and Modeling

### 2.1. Preparation of the Material and Characterization

**Figure 1:**
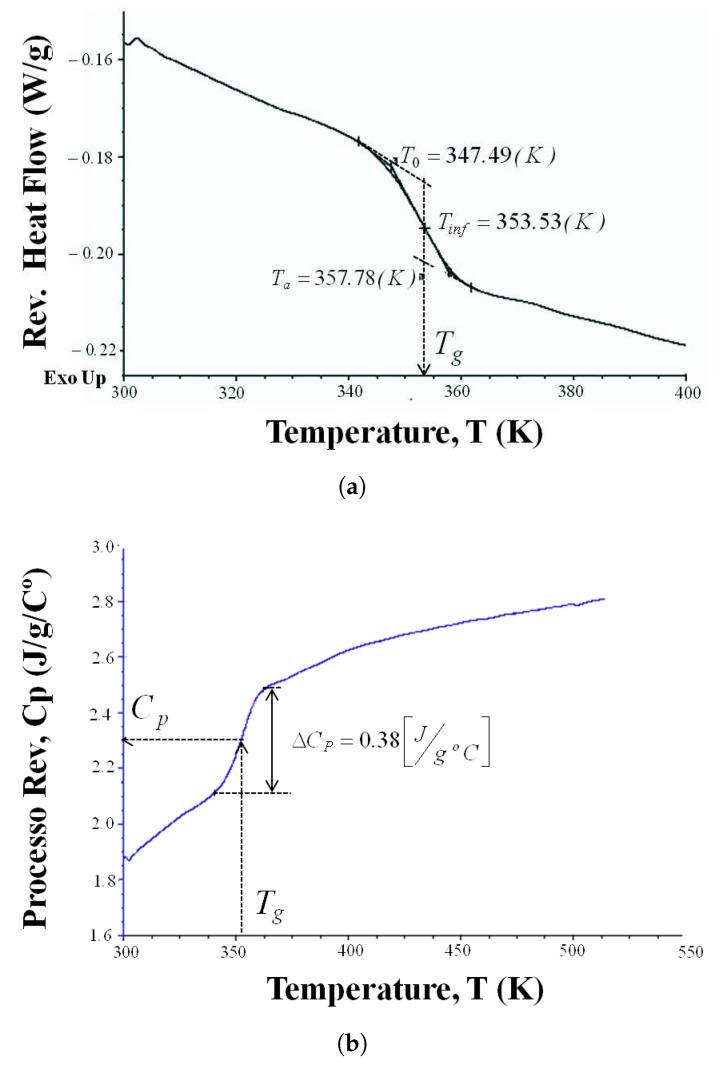
Results from modulated differential scanning calorimeter for the epoxy resin, (**a**) variation of the heat flow to determine the glass transition temperature $T_{g}$ and (**b**) derivation of the heat capacity, $C_{p}$.

The material used in this study is an epoxy resin RTM (Resin Transfer Molding), which is prepared from pure Bisphenol A epoxy resin and a polyamine liquid hardener, Isophoronnediamine (produced by Gurit). The weight ratio of the two constituents is 100:26 resin/hardener. Once mixed, these two constituents are cast into a preheated mold. The mixture is stored at room temperature for 22 h followed by a curing for 7 h at 65 ${}^{\circ }$C. The density of the cured system is 1.121 gr/cm${}^{3}$. Differential scanning calorimetry measurements (MDSC) are performed in a MDSC-Q20 (from TA Instruments). The MDSC curves are obtained with a sample of 3.5 mg (±0.1 mg) and a heating rate 5 ${}^{\circ }$C/min. The runs are performed by heating the samples from 25 ${}^{\circ }$C up to 250 ${}^{\circ }$C. From heat flow–temperature curves (see Figure 1), the glass transition temperature, $T_{g}$ (Figure 1a) and heat capacity, $C_{p}$ (Figure 1b), are extracted. The measurements result in $T_{g}=80.5$ ${}^{\circ }$C and $C_{p}=2300$ J/kg/${}^{\circ }$C.

The Poisson ratio ν and the shear modulus *G* are derived from measurements of the bulk and shear velocities with an ultrasonic setup (USM-25), which results in ν=0.38 and G=1.14 GPa (corresponding to a Young’s modulus of E=3.2 GPa).

### 2.2. Mechanical Response under Uniaxial Compression

To investigate the mechanical response of the epoxy, cylindrical specimens from an epoxy bulk with a 10 mm diameter and 10 mm height are prepared. In the compression tests, a boron nitride (NB) powder is laid between the samples and the plates of the test device to ensure a uniform uniaxial compression and prevent any barreling effect. In practice, no noticeable barreling is observed up to large deformations. Uniaxial compression tests are used to investigate the bulk response up to large strains (up to 100%) as failure is prevented and postponed when a hydrostatic stress prevails. These are conducted on the epoxy at room temperature on an Instron servo-hydraulic machine model 8801. A linear variable displacement transducer (LVDT) is used to measure the axial deformation of the specimen. The prescribed strain rate ranges from $1\times 10^{-5}\;s^{-1}$ to $1\times 10^{-1}\;s^{-1}$.

**Figure 2:**
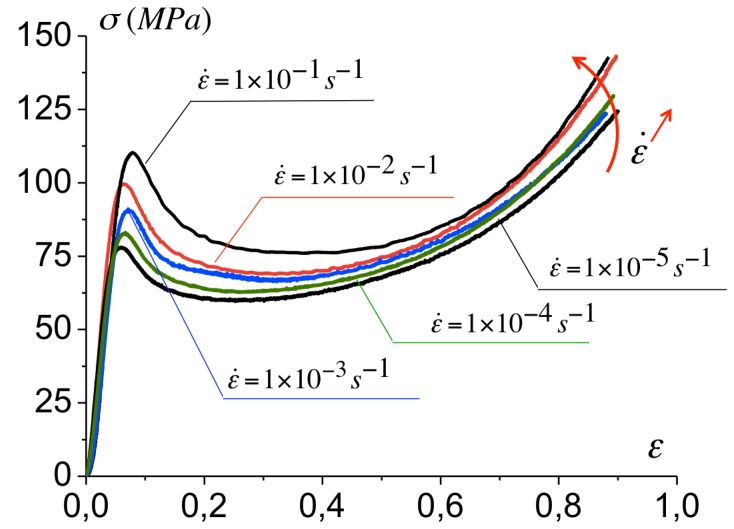
Compressive stress-strain curves for Epoxy resin RTM for a strain rate varying from $1\times 10^{-5}\;s^{-1}$ to $1\times 10^{-1}\;s^{-1}$ (the symbol ‘,’ is used for the decimal point).

The variation of the Cauchy stress with the axial logarithmic strain at various strain rates are reported in Figure 2. The Cauchy stress is calculated from the measure of the resulting force over the sample’s current section, under the assumption of an isochoric deformation. A rate-dependent yield stress is observed followed by a softening and hardening at continued deformation. For a strain rate varying from $1\times 10^{-5}\;s^{-1}$ to $1\times 10^{-1}\;s^{-1}$, the yield stress varies from 75 MPa to 112 MPa. A strain softening is observed upon yielding with a stress drop the magnitude of which is comparable for all strain rates. At continued deformation, a hardening takes place. The related stress increase is observed for the same strain range for all strain rates. Qualitatively, the response of the epoxy under uniaxial compression is comparable to observations in glassy polymers (see [7] in PMMA for instance). However, no thermal effects influencing the hardening part are observed in the epoxy, in contrast to observations reported by [8] in PMMA, and in general in glassy polymers. Although thermal effects can originate from the plastic conversion into heat, these induce minor or no effects on the softening and hardening in the epoxy under consideration. In the next section, the background of the model to describe the bulk response is presented.

### 2.3. Constitutive Model for the Epoxy Resin RTM

Based on the previous observations, we borrowed an elastic-viscoplastic model that captures the softening upon yielding followed by progressive orientation hardening initially developed for glassy polymers. The formulation proposed by Boyce et al. [9] later improved by Wu and van der Giessen [10] is adopted here since this enables a realistic description of the stress-strain fields. There is no specific physical motivation for using this model and the other formulation as proposed recently by Poulain et al. [11] could be used.

The elastic part of the deformation gradient is assumed to remain small compared to the plastic one so that the total strain rate D can be decomposed in the sum of the elastic and plastic parts with $D=D^{e}+D^{p}$. Prior to the yield stress, visco-elastic effects can induce a non- linear response. These are not considered explicitly but their effect on the mechanical response is accounted for by using a secant Young’s modulus instead of that derived from ultrasonic measurements of the elastic wave velocities. Its value is estimated from uniaxial compression tests and an average value for different strain rates of $E_{sec}=\sigma_{y}/\varepsilon_{y}$, with $\sigma_{y}$ the yield stress and $\varepsilon_{y}$ the corresponding yield strain is used. In view of these approximations, the hypo-elastic law is used to express the mechanical response as

(1) $$\overset{▽}{\sigma }=L^{e}:D^{e}=L^{e}:\left(D-D^{p}\right)$$

where $\overset{▽}{\sigma }$ is the Jaumann rate of the Cauchy stress, $L^{e}$ the fourth-order isotropic elastic moduli tensor in terms of secant Young’s modulus $E_{sec}$ and Poisson’s ratio ν which is in Cartesian components, $L_{ijkl}^{e}=\frac{E_{sec}}{2\left(1+\nu \right)}\left[\left(\delta_{ik}\delta_{jl}+\delta_{il}\delta_{jl}\right)+\frac{2\nu }{1-2\nu }\delta_{ij}\delta_{kl}\right]$, with $\delta_{ij}$ the Kronecker delta. Within the elastic-viscoplastic framework used here, the plastic strain rate is

(2) $$D^{p}={\dot{\gamma }}^{p}\frac{{\bar{\sigma }}^{\prime }}{\sqrt{2\tau }}$$

with ${\dot{\gamma }}^{p}\left(\tau ,T\right)$ the equivalent shear strain rate which is temperature and stress dependent, and $\tau =\sqrt{{\bar{\sigma }}^{\prime }\cdot {\bar{\sigma }}^{\prime }/2}$ the equivalent effective shear stress. In (2), the tensor ${\bar{\sigma }}^{\prime }$ corresponds to the deviatoric part of the driving stress which is the difference between the applied Cauchy stress σ and the back stress b as $\bar{\sigma }=\sigma -b$. The back stress tensor is due to the entropic back forces generated by the deformation of the polymer network during the plastic deformation and will be defined in the sequel. The equivalent plastic shear strain rate ${\dot{\gamma }}^{p}$ is taken according to [12] as

(3) $${\dot{\gamma }}^{p}={\dot{\gamma }}^{0}\exp\left[-\frac{As_{0}}{T}\left(1-{\left(\frac{\tau }{s_{0}}\right)}^{\frac{5}{6}}\right)\right]$$

in which *A* and ${\dot{\gamma }}^{0}$ are material parameters and *T* the absolute temperature. The athermal shear strength is $s_{0}=\frac{0.077G}{1-\nu }$, with *G* the shear modulus at high frequency and ν the Poisson ratio. A pressure dependence of the plastic shear strain rate is not accounted for in the present study as no quantification of this effect is available for the material under consideration. Following Boyce et al. [9], intrinsic softening is accounted for with the definition of an internal variable *s* which varies from $s_{0}$ to a steady state $s_{ss}$ at continued plastic deformation. The internal law

(4) $$\dot{s}=h\left(1-\frac{s}{s_{ss}}\right){\dot{\gamma }}^{p}$$

governs its variation during deformation, with *h* a parameter controlling the rate of softening and $s_{ss}$ the steady state value of *s*. The progressive hardening due to increasing plastic deformation and induced molecular orientation results in a back stress tensor b which can be considered to be the development of internal stresses during the deformation. Its description is based on ideas borrowed from rubber elasticity [9,10,13]. The estimate of the back stress b used in our description is due to Wu and Van der Giessen [10] based on their analysis of the fully three-dimensional orientation distribution of molecular chains in a non-Gaussian network. Wu and Van der Giessen (1993) have shown that a good estimate of the back stress tensor b can be obtained with the following combination of the three-chain and eight-chain [14] models as

(5) $$b_{\alpha }=\left(1-\rho \right)b_{\alpha }^{3-ch}+\rho b_{\alpha }^{8-ch},$$

with $\rho =0.85\bar{\lambda }/\sqrt{N}$, $\bar{\lambda }=max\left(\lambda_{1}+\lambda_{2}+\lambda_{3}\right)$ the maximum plastic stretch and *N* the average number of segments between entanglements or rigid bonds. The limit stretch of a molecular chain is $\lambda_{max}=\sqrt{N}$. The principal back stress components $b_{\alpha }^{3-ch}$ and $b_{\alpha }^{8-ch}$ are given by

(6) $$b_{\alpha }^{3-ch}=\frac{1}{3}C^{R}\sqrt{N}\lambda_{\alpha }L^{-1}\left(\frac{\lambda_{\alpha }}{\sqrt{N}}\right)$$

(7) $$b_{\alpha }^{8-ch}=\frac{1}{3}C^{R}\sqrt{N}\frac{\lambda_{\alpha }^{2}}{\lambda_{c}}L^{-1}\left(\frac{\lambda_{\alpha }}{\sqrt{N}}\right)$$

where *L* denotes the Langevin function defined as $L\left(\beta \right)=\coth\left(\beta \right)-\frac{1}{\beta }$. The material parameter $C^{R}$ governs the initial hardening modulus, taken as $C^{R}=nk_{B}T$, *n* being the cross-link density, $k_{B}$ the Boltzmann constant and *T* the temperature.

### 2.4. Identification of Bulk Parameters for the Epoxy Resin

**Figure 3:**
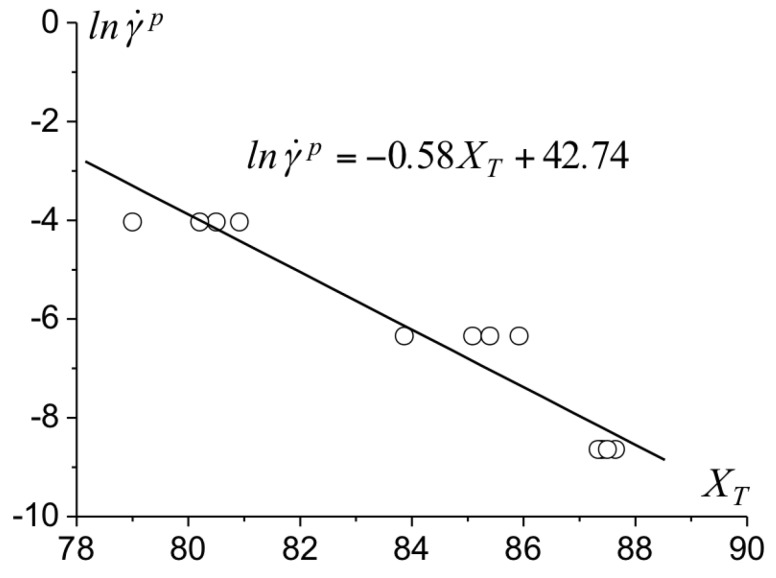
Variation of the plastic shear strain rate with the generalized variable $X_{T}$ for strain rates between $1\times 10^{-5}\;s^{-1}$ and $1\times 10^{-3}\;s^{-1}$, the circles correspond to the experimental measurements.

The parameters involved in the bulk constitutive law of the epoxy are identified from uniaxial compression tests at various loading rates as in [7]. The stress-strain response prior to yielding is approximated by a linear secant modulus $E_{sec}$ of which average value of $\sigma_{y}/\varepsilon_{y}$ at the yield stress results in $E_{sec}=1395$ MPa. The athermal yield stress $s_{0}$ is derived from $s_{0}=\frac{0.077G}{1-\nu }=140$ MPa. The strain rate and temperature dependence of the initial yield stress involves the parameters *A* and ${\dot{\gamma }}^{0}$ in (3). At the yield stress and prior to softening, the plastic strain rate ${\dot{\gamma }}^{p}$ equals the prescribed strain rate $\dot{\gamma }=\sqrt{3}\dot{\varepsilon }$, $\dot{\varepsilon }$ being the uniaxial compression strain rate. The shear stress is $\tau_{y}=\sigma_{y}/\sqrt{3}$ at yielding. Equation (3) can be reformulated as ${\dot{\gamma }}^{p}={\dot{\gamma }}^{0}\exp\left[-\left(A/T\right)X_{T}\right]$, in which $X_{T}=s_{0}\left[1-{\left(\tau_{y}/s_{0}\right)}^{5/6}\right]$. The parameters ${\dot{\gamma }}^{0}$ and *A* are derived from the linear variation of $\ln{\dot{\gamma }}^{p}$ with $X_{T}$ at room temperature reported in Figure 3.

**Figure 4:**
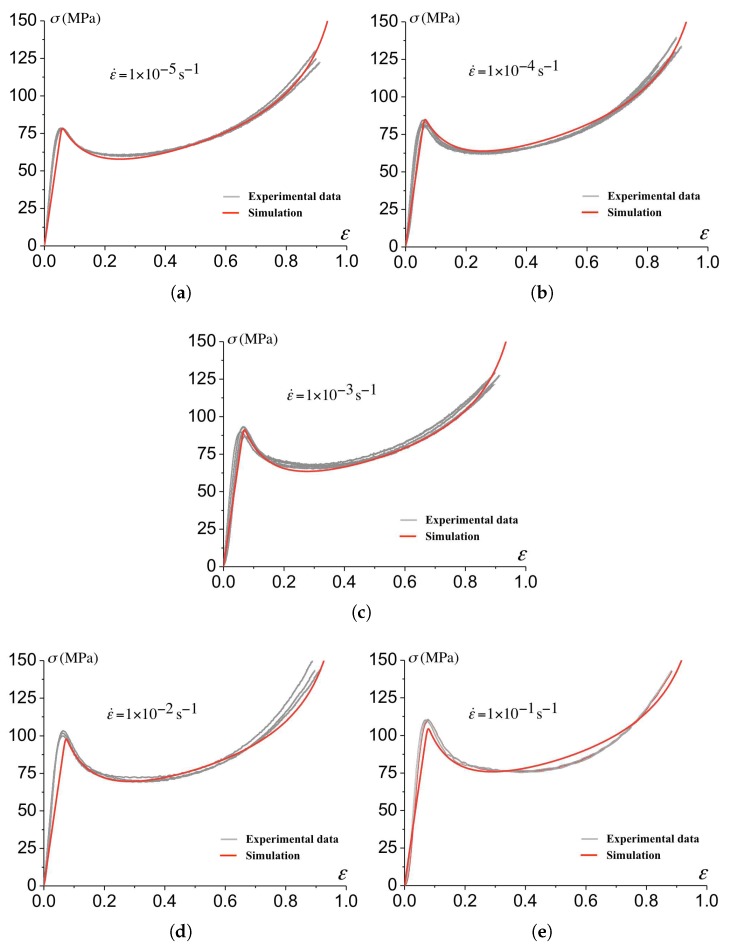
The stress-strain relationship predicted from the constitutive model (Section 2.3) using the parameters reported in Table 1 for (**a**) $1\times 10^{-5}\;s^{-1}$, (**b**) $1\times 10^{-4}\;s^{-1}$, (**c**) $1\times 10^{-3}\;s^{-1}$, (**d**) $1\times 10^{-2}\;s^{-1}$ and (**e**) $1\times 10^{-1}\;s^{-1}$.

The parameters for the description of the post-yield response (softening and hardening) are derived from the best fit between the simulations of the uniaxial compression tests and the experimental data. The corresponding fit is shown in Figure 4 and the parameters are reported in Table 1.

Figure 4 shows the stress-strain predictions and the measurements for a strain rate varying between $1\times 10^{-5}\;s^{-1}$ to $1\times 10^{-1}\;s^{-1}$ with good agreement up to 100% of deformation. The calculation are performed under the assumption of isothermal conditions.

The bulk parameters are identified under the assumption of isothermal conditions, which may be questionable for the largest strain rates. A temperature variation during the compression tests can be observed for strain rates larger than $\dot{\varepsilon }=1\times 10^{-2}\;s^{-1}$. Calculations under the assumption of adiabatic conditions were carried out with $\rho c\dot{T}={\bar{\sigma }}^{\prime }D^{p}$ with $\rho =1200\;{\mathrm{kg}/m}^{-3}$, the heat capacity c=2.3J/g/K, and ${\bar{\sigma }}^{\prime }D^{p}$ the effective plastic energy dissipation rate per unit volume. The related temperature increase can influence the plastic strain rate (See Equation (3)) and the plastic response. However, no influence on the stress-strain response is found in the present case, as the network is not modified by the temperature variation, unlike glassy polymers.

The parameters identified for the description of the bulk response are reported in Table 1.

## 3. Modeling of the Fracture Process with a Rate-Dependent Cohesive Model: Fracture Tests, Experiments and Simulations

In this section, an experimental investigation of fracture of the epoxy resin is presented. Machining a sharp crack as recommended by the ESIS-TC4 technical committee without generating plastic deformation is not practically doable. The preparation of blunted notches that are machined with a cutting tool may induce initial stresses around the notch or generate a temperature increase that modifies the bulk constitutive response locally. If this happens, as these features are not accounted for in the simulations, the comparison between the predictions and the experimental measurements are not straightforward. Thus, we prepared the notches under a flow of fresh air. We did verify after the machining that under a crossed polarizer the light transmitted is uniform, even around the notch region. No thickness reduction is observed during this machining. For each geometry, the variation of the toughness derived from the measure of the force at rupture with the loading rate is reported. These data will be used for the identification of the cohesive parameters and a validation of the predicted load level for fracture will be presented.

### 3.1. Preparation of the Samples

Plates of epoxy resins with a thickness of 10 mm are processed. Parallelepipedal samples for four-point bending tests are prepared and blunted notched machined with a radius varying between 0.05–0.5 mm.

Single blunted notch specimens (SENB) are loaded under a four-point bending to ensure mode I. The variation of the fracture toughness with the loading rate is reported in the sequel.

### 3.2. Fracture Response under Mode I

Fracture tests are carried out using an Instron servo-hydraulic tensile test machine, in which a force rate is prescribed thus corresponding to a constant rate of the stress intensity factor (SIF) ${\dot{K}}_{I}=dK_{I}/dt$ according to [15],

(8) $${\dot{K}}_{I}=\dot{\sigma }\sqrt{\pi a}f\left(\frac{a}{W}\right).$$

The force control allows us to set a constant value of $\dot{F}$ and thus, a loading rate constant stress rate $\dot{\sigma }$,

(9) $$\dot{\sigma }=\frac{3}{2}\times \frac{\left(S_{1}-S_{2}\right)}{BW^{2}}\dot{F}.$$

In Equation (8), *a* is the crack length and *W* the specimen width, the geometry function for the four-point bending configuration is [15],

(10) $$f\left(\frac{a}{W}\right)=1.122-1.4\left(\frac{a}{W}\right)+7.33{\left(\frac{a}{W}\right)}^{2}-13.08{\left(\frac{a}{W}\right)}^{3}+14{\left(\frac{a}{W}\right)}^{4}.$$

**Figure 5:**
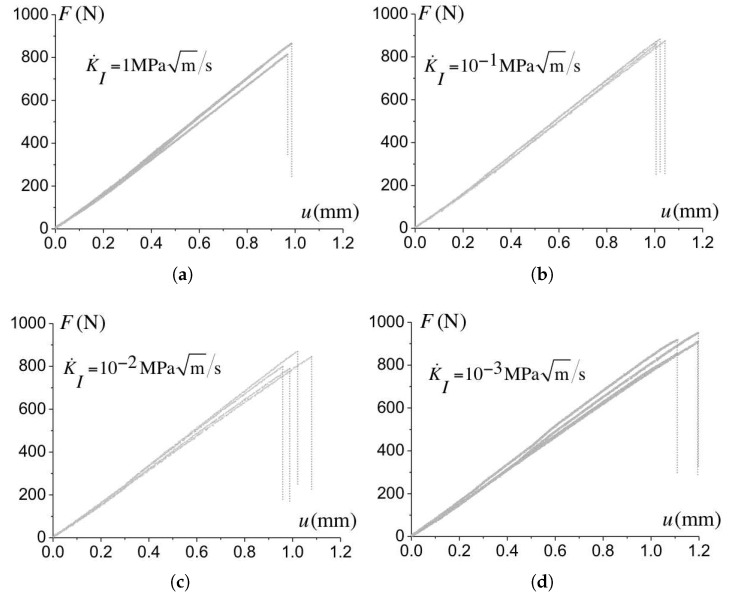
Force versus displacement obtained for the geometry with a notch radius $r_{t}=0.15$ mm recorded for various loading rates as: (**a**) ${\dot{K}}_{I}=1$ MPa$\sqrt{m}/s$; (**b**) ${\dot{K}}_{I}=10^{-1}$ MPa$\sqrt{m}/s$; (**c**) ${\dot{K}}_{I}=10^{-2}$ MPa$\sqrt{m}/s$ and (**d**) ${\dot{K}}_{I}=10^{-3}$ MPa$\sqrt{m}/s$.

The tests are conducted under ambient conditions (temperature and humidity) for loading rates ranging from ${\dot{K}}_{I}\;=\;10^{-3}$ MPa$\sqrt{m}/s$ to ${\dot{K}}_{I}\;=\;1$ MPa$\sqrt{m}/s$. The force versus displacement curves for the geometry with $r_{t}=0.15$ mm are reported in Figure 5. The onset of crack propagation occurs when a maximum force is attained, thus defining the critical force and related toughness. No variation of the compliance prior to failure is observed.

**Figure 6:**
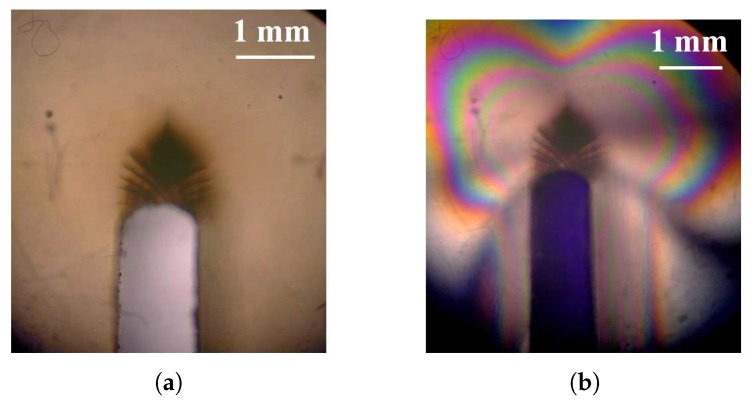
(**a**) Experimental observation of shear bands around a blunt notch in Epoxy resin, (**b**) crossed polarizer observations of the notch tip.

Experiments have been carried out for the other notch radii $r_{t}=0.05$ mm and $r_{t}=0.11$ mm. The corresponding force–displacement curves show a linear variation up to failure. However, a plastic zone in the form of shear bands is observed at the notch tip (see Figure 6). The observed plastic region with shear bands is comparable to that reported by Ishikawa et al. [16] in polycarbonate. The plastic zone is similar to Hill slip bands [17] and to those analyzed in detail by Lai and der Giessen [18] with account for a realistic constitutive law for polymers. Noticeable plasticity precedes fracture so that the failure is ductile. This also indicates that the cross-link density is low for the epoxy under consideration.

**Figure 7:**
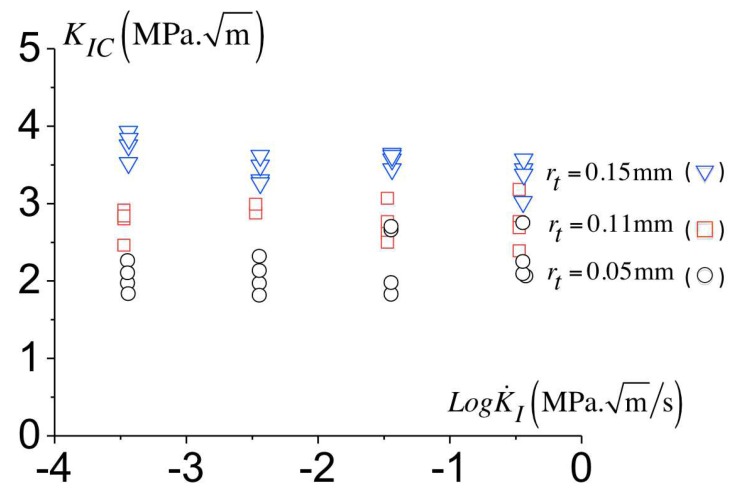
Variation of the critical toughness for different notch radii with the loading rates.

Figure 7 presents the variation of the toughness with the loading rate for the configuration with a notch radius of 0.05 mm, 0.11 mm and 0.15 mm. For each geometry and at a given loading rate, four tests are performed and reported. The variation of the toughness with the loading rate shows a modest decrease of the load level for fracture with the loading rate $\dot{K}$ increasing. The rate dependence can originate from (i) the bulk viscoplastic response but also (ii) from a rate dependent fracture process, as shown by [6,7] for glassy polymers. A rate-dependent cohesive model for failure is considered to (i) describe the fracture mechanism and (ii) predict the material’s toughness, which is presented in the next section.

### 3.3. Rate-Dependent Cohesive Zone Model for Failure in Epoxy Resin

**Figure 8:**
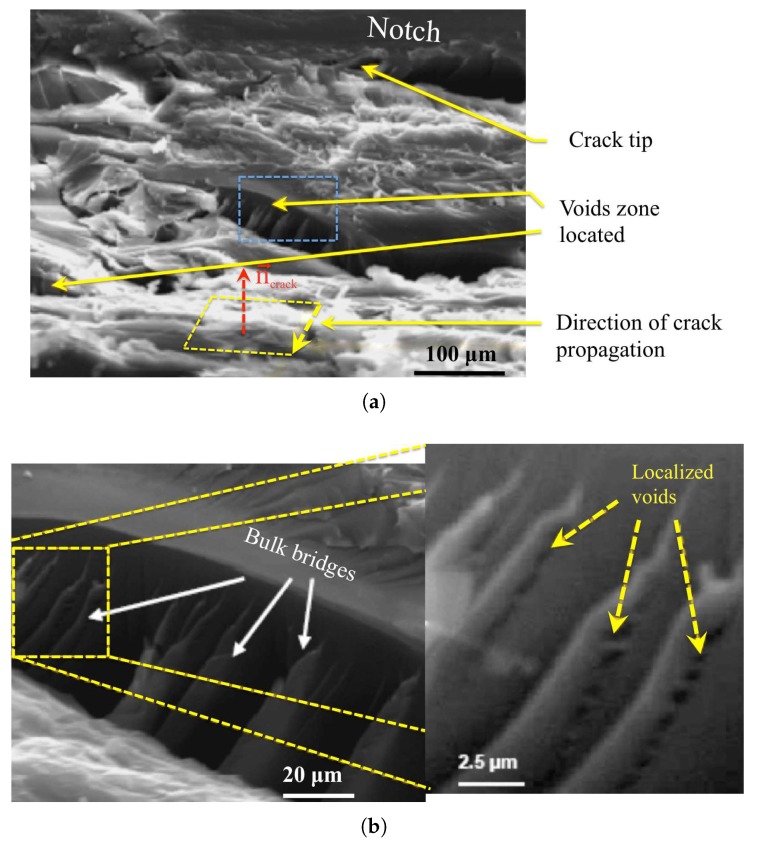
SEM observation of one fractured sample ($r_{t}=0.05$ mm, ${\dot{K}}_{I}=10^{-3}$ MPa$\sqrt{m}/s$), (**a**) Zone ahead from the notch, the box in blue shows the voids located and (**b**) the box zoomed.

In polymer thermosets with a high cross-link density, Kinloch [2] considers that craze fibrillation is not likely to occur. However, the existence of crazes similar to that observed in amorphous polymers is still under debate. Lilley and Holloway [3] reports some evidence of crazes and later “croids” consisting of void formation and growth by localized plastic deformation have been reported by Yee and Pearson [19]. More recently, Kanchanomai et al. [5] observed the fracture surface of an epoxy and evidenced an arrow of voids in between two parallel surfaces. This latter observation looks like a craze structure. Based on this later reference, SEM (Scanning Electron Microscopy) observations have been conducted in one of the fractured samples ($r_{t}=0.05$ mm, ${\dot{K}}_{I}=10^{-3}$ MPa$\sqrt{m}/s$). The observation of the fracture surface is presented in Figure 8. The normal to the fracture plane is reported. The roughness indicates a ductile mechanism that involves the formation of voids in elongated regions, similarly to Kanchanomai et al. [5] observations. Therefore, we assume that localized plasticity with the formation and growth of voids is involved in the failure mechanism. As plasticity is rate dependent, we adopt a rate dependent cohesive model to mimic mechanically the fracture mechanism, which is borrowed to Tijssens et al. [20] initially develop to describe crazing in glassy polymer. We do not claim that crazing takes place here. We only assume that failure involves a localized rate-dependent process.

In glassy polymers failure by crazing is assumed to proceed in three stages: (i) initiation for a local critical stress state and related cavitation of the material, (ii) widening with the development of a web of fibrils, (iii) breakdown of the fibrils and creation of a crack locally, for a critical craze opening. These three stages can be incorporated in a cohesive model as used in Saad-Gouider et al. [7] who have shown that the use of a rate-dependent cohesive zone is necessary to capture the variation of the fracture toughness variation with loading rate even in brittle materials such as PMMA. We recall the basis of the rate-dependent model adopted in the present study and the formulation of the traction-opening law which describes such a possible rate dependence.

### 3.4. Description of the Failure Process in Low Cross-Link Epoxy

The failure process is assumed to proceed in three stages with (i) nucleation of voids for a critical stress state, (ii) the growth of the localized damage zone with the thickening of the cohesive surface up to (iii) a critical opening corresponding to the nucleation of a crack.

#### 3.4.1. Initiation

The failure mechanisms, likely by the nucleation of voids, is triggered when a local critical traction along the normal of the cohesive surface is overcome as $\sigma_{n}\geq \sigma_{n}^{cr}$.

#### 3.4.2. Damage Zone and Cohesive Thickening

Following [20], the thickening of the cohesive zone is described with a viscoplastic formulation for the separation of the cohesive surfaces with the opening rate

(11) $${\dot{\Delta }}_{n}^{c}={\dot{\Delta }}^{0}\exp\left[\frac{-A^{c}\sigma^{c}}{T}\left(1-\frac{\sigma_{n}}{\sigma^{c}}\right)\right],$$

in which ${\dot{\Delta }}^{0}$ a pre-exponential term that has the dimension of a velocity, $A^{c}$ accounts for the temperature dependence and $\sigma^{c}$ is a characteristic athermal stress. The thickening rate ${\dot{\Delta }}_{n}^{c}$ represents the opening of the cohesive surface subjected to a normal traction $\sigma_{n}$.

#### 3.4.3. Crack Nucleation

The thickening of the cohesive surface continues up to the critical opening $\Delta_{n}^{cr}$ that corresponds to the nucleation of a crack locally, $\Delta_{n}^{cr}$ being a material parameter. The cohesive formulation is incorporated through the traction-opening relationship

(12) $${\dot{\sigma }}_{n}=k_{n}\left({\dot{\Delta }}_{n}-{\dot{\Delta }}_{n}^{c}\right),$$

with ${\dot{\Delta }}_{n}$ the normal opening rate of the cohesive surfaces, ${\dot{\Delta }}_{n}^{c}$ the thickening rate according to (11) and $k_{n}$ a stiffness large enough to ensure ${\dot{\Delta }}_{n}\approx {\dot{\Delta }}_{n}^{c}$ during the thickening. Prior to initiation, (12) reduces to ${\dot{\sigma }}_{n}=k_{n}{\dot{\Delta }}_{n}$ in which the stiffness $k_{n}$ must be “infinitely” large to ensure the continuity of the displacement fields. When the critical opening is attained, a crack nucleates locally which is accounted for with a vanishing traction on the corresponding surfaces. Details of the numerical aspects are presented in [6,20].

**Figure 9:**
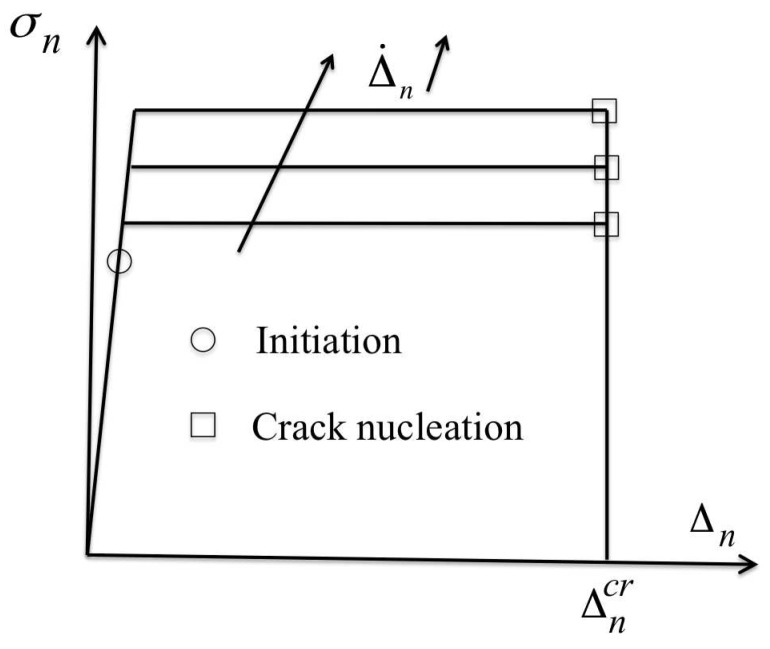
Traction-opening law of the cohesive model for fracture.

In Figure 9, the response of the cohesive model is shown schematically. Three parts are distinguished with a stiff response prior to initiation of the failure mechanism followed by a plateau with a constant traction of which magnitude is governed by the condition ${\dot{\Delta }}_{n}\approx {\dot{\Delta }}_{n}^{c}$, that result in a rate-dependent response. The thickening of the cohesive surfaces continues up to the critical opening $\Delta_{n}^{cr}$ when failure takes place. This formulation can be interpreted as a rate-dependent Dugdale model.

No contribution to damage from the tangential mode is considered so that ${\dot{\sigma }}_{t}=k_{t}{\dot{\Delta }}_{t}$, with $k_{t}=k_{n}$. The motivation for this choice is that no experimental data are available for its characterization. Practically, once breakdown takes place, $\sigma_{t}$ and $k_{t}$ are also set to zero.

The calibration of the cohesive zone parameters is derived from fracture tests under quasi-static loading conditions, and will be presented in the sequel.

### 3.5. Problem Formulation for Mode I Fracture

**Figure 10:**
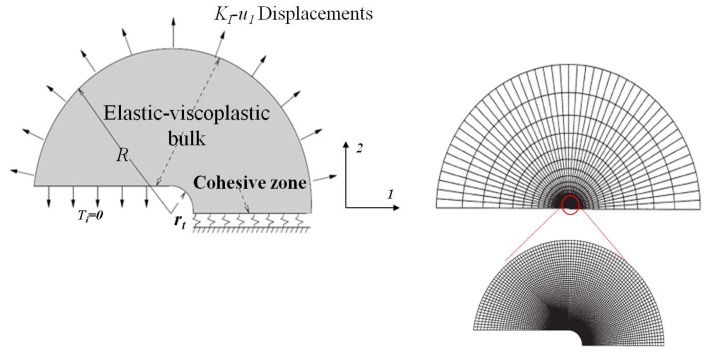
Description of the mode I small-scale yielding problem, the mesh and the zoom of the mesh around the crack.

Simulations are performed with a finite element analysis of mode I fracture depicted in Figure 10. Bulk plasticity and failure are assumed to be confined around the crack tip so that the small-scale yielding framework is allowed. The boundary layer approach is used to investigate the mode I under plane strain conditions.

A cohesive surface is laid out ahead of the crack along the symmetry plane, where initiation of failure is most likely. The remote region consists in a circular arc along which the $K_{I}$ displacement fields (see Figure 10) are prescribed as

(13) $$u_{1}=2\left(1+\nu \right)\frac{K_{I}}{E_{sec}}\sqrt{\frac{r}{2\pi }}\cos\frac{\theta }{2}\left[2-2\nu -{\left(\cos\frac{\theta }{2}\right)}^{2}\right]$$

(14) $$u_{2}=2\left(1+\nu \right)\frac{K_{I}}{E_{sec}}\sqrt{\frac{r}{2\pi }}\sin\frac{\theta }{2}\left[2-2\nu -{\left(\cos\frac{\theta }{2}\right)}^{2}\right],$$

in which the secant Young modulus $E_{sec}$ is used. The constitutive law for the bulk material is elastic-viscoplastic (see Section 2.3).

A total Lagrangian description is adopted and the rate form of the virtual work is used to solve the problem incrementally (see [6,20,21] for a detailed presentation of numerical aspects).

Convected coordinates are used to account for large deformation that allows the description of the problem in the initial configuration.

### 3.6. Calibration of the Cohesive Model

The calibration of the cohesive model consists in the identification of the critical traction $\sigma_{n}^{cr}$ for the onset of the mechanism underlying failure, the parameters (${\dot{\Delta }}^{0}$, $A^{c}$ and $\sigma^{c}$) in (11) describing the fracture mechanism kinetics and the critical opening $\Delta_{n}^{cr}$. This latter value is of the order of some microns in amorphous polymers [22] and no direct measurement is available for thermosets. Based on Döll data, the critical opening is first set to $\Delta_{n}^{cr}=3$ μm and its influence on the toughness predictions will be investigated by varying $\Delta_{n}^{cr}$ from 1 μm to 5 μm. The fracture mechanism proceeds when the cohesive traction, $\sigma_{n}\geq \sigma_{n}^{cr}$ with $\sigma_{n}^{cr}=50$ MPa. This value is smaller than the magnitude of the traction for which thickening of the cohesive surfaces is noticeable (see in Figure 9), and is of minor importance for the toughness’ predictions. As in Saad-Gouider et al. [7], we choose a temperature dependence for the rate-dependent thickening rate in (11) identical to the bulk so that $A^{c}=A=173$ K/MPa. Thus, the parameters (${\dot{\Delta }}^{0}$, $\sigma^{c}$) from (11) remain to be identified. From the uniaxial tests, the maximum stress is closed to 140 MPa and this value is chosen for $\sigma^{c}$ in (11). Thus, only the pre-exponential parameter ${\dot{\Delta }}^{0}$ needs to be calibrated.

We can notice that changing $\sigma^{c}$ would result in a change in ${\dot{\Delta }}^{0}$ but does not influence the kinetics of the cohesive surface as Equation (11) can be rearrange as

(15) $${\dot{\Delta }}_{n}^{c}={\dot{\Delta }}^{0}\left(\exp\frac{-A^{c}\sigma^{c}}{T}\right)\times \exp\left(\frac{A^{c}\sigma_{n}}{T}\right).$$

**Figure 11:**
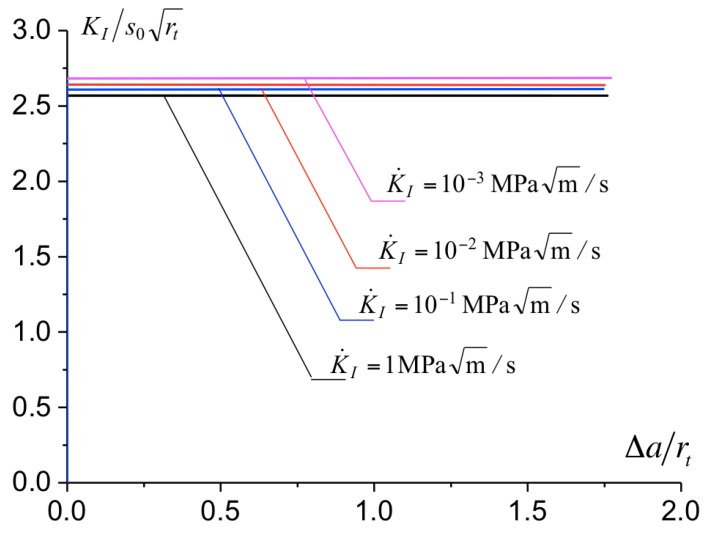
Resistance curve in term $K_{I}$ versus the length of the notch for different loading rates.

**Figure 12:**
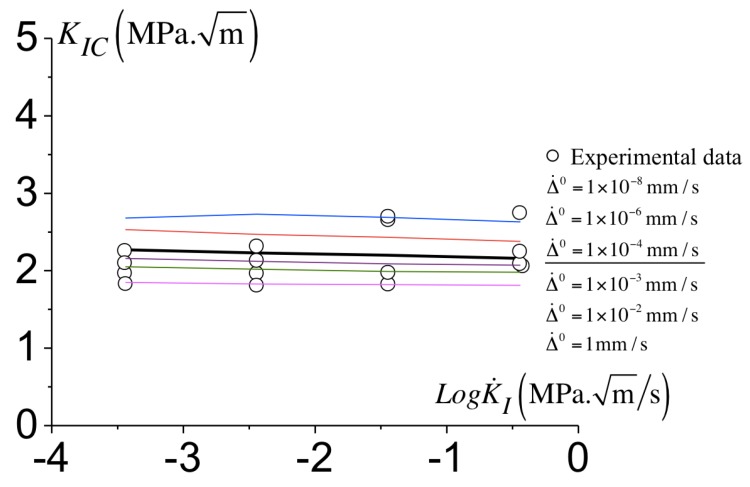
Predicted toughness $K_{IC}$ with the loading rate for different ${\dot{\Delta }}^{0}$ of (11). The experimental data corresponding to $r_{t}=0.05$ mm.

By fixing $A^{c}$ and $\sigma^{c}$ in (11), ${\dot{\Delta }}^{0}$ is adjusted from the best fit between the toughness predicted in the simulation and the experimental measurements. How the toughness is extracted from the simulation is presented in Figure 11 where the load level in terms of $K_{I}$ (normalized by $s_{0}\sqrt{r_{t}}$) is reported against the crack growth Δa (normalized with the notch radius $r_{t}$). At the onset of failure and during crack propagation, a constant load level is observed. The value of $K_{I}$ at this plateau corresponds to the predicted toughness $K_{IC}$. For the calibration of the cohesive parameters, the geometry with a notch radius equal to $r_{t}=0.05$ mm is used. The comparison between the predicted toughness and the experimental measurements is presented in Figure 12. Various values for ${\dot{\Delta }}^{0}$ has been considered and the best fit between the predictions and measurements is obtained for ${\dot{\Delta }}_{0}=1\times 10^{-4}$ mm/s. Following a trial and error procedure, we identify ${\dot{\Delta }}^{0}$ ranging from 1 mm/s to $1\times 10^{-8}$ mm/s of which predictions of $K_{IC}$ are within the experimental scatter (see Figure 12). The thickest line in Figure 12 corresponds the toughness predictions with ${\dot{\Delta }}_{0}=1\times 10^{-4}$ mm/s which best captures the toughness variations of the toughness $K_{IC}$ with the loading rate. The parameters identified for the cohesive model are reported in Table 2.

**Figure 13:**
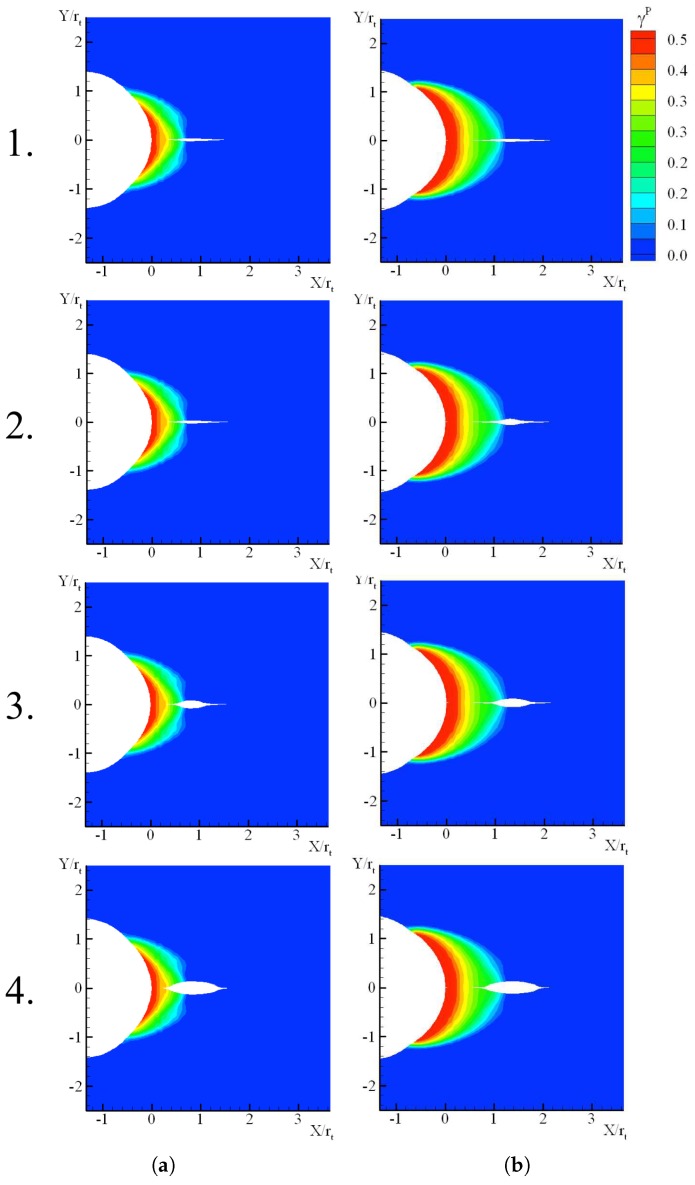
Distribution of the cumulated plastic shear strain $\gamma^{p}$ for (**a**) ${\dot{K}}_{I}=1$ MPa$\sqrt{m}/s$ and (**b**) ${\dot{K}}_{I}=1\times 10^{-3}$ MPa$\sqrt{m}/s$. (**1**) Corresponds to the cohesive surfaces thickening, with its onset at the tip of the plastic zone, (**2**) crack nucleation when $\Delta_{n}^{c}=\Delta_{n}^{cr}$ is found first along the cohesive surfaces and (**3**,**4**) show how the crack propagates.

In Figure 13, the initiation of the failure mechanism is preceded by noticeable plasticity in the bulk. Its location is at the tip of the plastic zone, where the stress concentrates due to the plastic incompatibility. The cohesive surface thickening continues up to (2) the nucleation of a crack. Crack propagation then develops along the direction to the notch tip and ahead the crack. In Figure 13, we observe that for the largest and smallest loading rates considered, plasticity precedes failure so that ductile fracture takes place as observed in Figure 6. The size of the plastic zone for ${\dot{K}}_{I}=1$ MPa$\sqrt{m}/s$ is approximately 40% smaller than that observed for ${\dot{K}}_{I}=1\times 10^{-3}$ MPa$\sqrt{m}/s$. However, this variation results in a modest decrease of the toughness (see Figure 12). It is worth noting that the present methodology allows for a detailed analysis of the failure process, as it is in general available in a local approach of fracture. The local observation of the crack initiation ahead of the notch tip is realistically captured by the model.

### 3.7. Influence of the T-Stress on the Toughness

**Figure 14:**
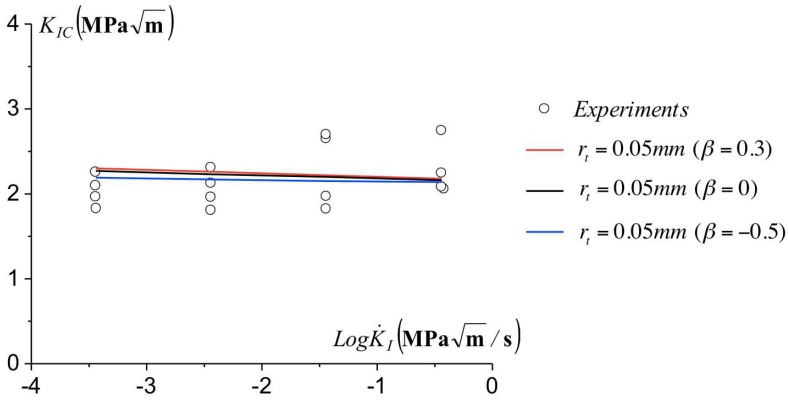
Influence of the T-stress on the toughness $K_{IC}$ with the loading rate for different ${\dot{\Delta }}^{0}$ of (11). The experimental data corresponding to $r_{t}=0.05$ mm.

When plasticity precedes failure, the magnitude of the plastic region is known to depend markedly on the T-stress (see Larsson and Carlsson [23], Rice [24], Leevers and Radon [25]), for metals and in general for elastic-plastic material, with no softening. Thus, the comparison between the small-scale yielding predictions and the experimental data reported in the previous section may be questionable as the tests are conducted for SENT (Single Edge Notch Tension), for which the magnitude of the T-stress can be noticeable. We present additional calculations for $r_{t}=0.05$ mm and the loading rates ranging from ${\dot{K}}_{I}=1\times 10^{-3}$ MPa$\sqrt{m}/s$ to ${\dot{K}}_{I}=1$ MPa$\sqrt{m}/s$ in which the T-stress is accounted for. Within a boundary layer approach, the prescribed displacement (13) and (14) are modified as

(16) $$u_{1}=\left(1+\nu \right)\frac{K_{I}}{E_{sec}}\sqrt{\frac{r}{2\pi }}\cos\left(\frac{\theta }{2}\right)\left(3-4\nu -\cos\theta \right)+T\frac{1-\nu^{2}}{E_{sec}}R\cos\theta$$

(17) $$u_{2}=\left(1+\nu \right)\frac{K_{I}}{E_{sec}}\sqrt{\frac{r}{2\pi }}\sin\left(\frac{\theta }{2}\right)\left(3-4\nu -\cos\theta \right)+T\frac{1-\nu^{2}}{E_{sec}}R\sin\theta$$

in which the magnitude of *T* is related to the biaxial ratio $\beta =\frac{T\sqrt{\pi a}}{K_{I}}$. We consider a large range for the biaxial ratio, as for a/W=0.5, ranges from β=−0.5 for Double Edge Notched Tension to β=0.3 for Single Edge Notch Bending. We report in Figure 14 the variation of the predicted toughness for β=−0.5.0 and 0.3.

We do not observe a noticeable influence of the magnitude of the T-stress in the toughness predictions. In the case of polymers which exhibit softening upon yielding, the notch-tip plasticity is heterogeneous as it proceeds in the form of shear bands, which cross along the notch plane. The stress generated at the shear bands intersection is likely to be larger than that related to the T-stress, in contrast to what is reported in metals and hardening elastic-plastic materials. Thus, no noticeable influence of the configuration and related T-stress is observed, as long as plasticity remains confined.

### 3.8. Validation

In order to validate the calibration of the cohesive parameters reported in Table 2, the predictions are compared to the experimental data for other geometries consisting of samples with notch radii of $r_{t}=0.11$ mm and $r_{t}=0.15$ mm.

**Figure 15:**
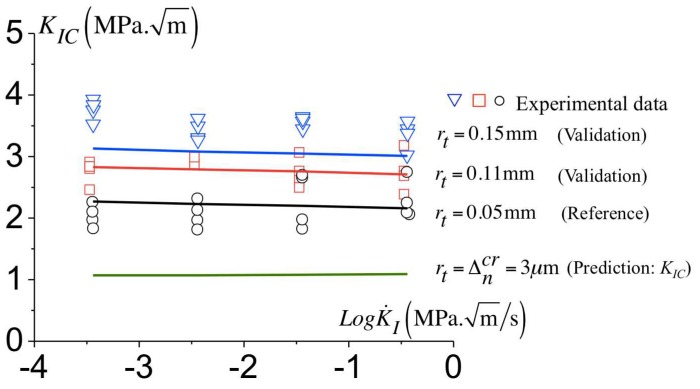
Variation of the critical toughness $K_{IC}$ for different notch radii at various loading rates, experimental data and predictions with the cohesive parameters presented in the Table 2.

Figure 15 shows the comparison between the predicted toughness to the measured one for three notch radii. The predicted toughness increases with the notch radius and are comparable to the experimental data. The model also predicts a modest decay of the toughness with the loading rate as found experimentally related to a little reduction in the amount of plasticity at the onset of failure. Since Figure 15 shows a good agreement between the predictions and the experimental data, a prediction of the minimum toughness for a sharp crack can be derived. To the end, an initial notch radius equal to the maximum opening $\Delta_{n}^{cr}$ is considered. The predicted toughness for a sharp crack results in $K_{IC}=1.08$ MPa$\sqrt{m}$, which appears constant over range of the loading rates investigated here.

### 3.9. Influence of the Critical Opening, $\Delta_{n}^{cr}$ on the Failure’s Predictions

**Figure 16:**
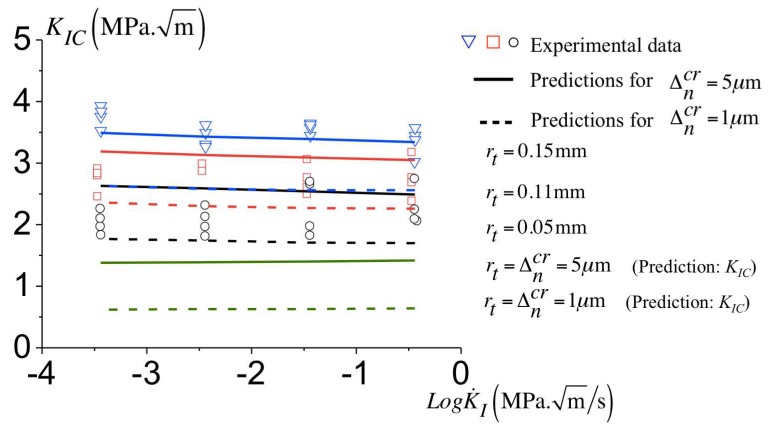
Variation of the critical $K_{IC}$ with different notch radii at various loading rates, experimental and predictions with $\Delta_{n}^{cr}=$1 μm and $\Delta_{n}^{cr}=$5 μm.

For the parameters identified for the cohesive model (see Table 2), we now evaluate how much the critical opening influences the predicted toughness. This is investigated by taking $\Delta_{n}^{cr}$ equal to 1 μm and 5 μm instead of $\Delta_{n}^{cr}=3$ μm. The variation of the predicted toughness for different configurations and loading rates is reported in Figure 16.

For the case of $\Delta_{n}^{cr}=1$ μm there is a small decrease of the predicted toughness compared to that found with $\Delta_{n}^{cr}=3$ μm. The predicted toughness for $\Delta_{n}^{cr}=1$ μm is smaller to the experimental data as the notch radius increases. The minimum toughness (for $r_{t}=\Delta_{n}^{cr}=1$ μm) is also derived, and appears constant over all loading rates and the value is about $K_{IC}=0.64$ MPa$\sqrt{m}$.

The Figure 16 also shows the variation of the toughness for all configuration using a critical opening $\Delta_{n}^{cr}=5$ μm. For this case, the predictions of the toughness presents a fairly good agreement with the experimental data as observed for the reference case ($\Delta_{n}^{cr}=3$ μm). The minimum predicted toughness (for $r_{t}=\Delta_{n}^{cr}=5$ μm) is almost constant for all loading rates configurations and the value is $K_{IC}=1.39$ MPa$\sqrt{m}$.

The value of $\Delta_{n}^{cr}$ has a noticeable influence on the predicted toughness for all notch radii under consideration here. The kinetics of the failure mechanism reported in Table 2 have been used for the prediction with $\Delta_{n}^{cr}$ ranging from 1 μm to 5 μm. When comparing the variation of the toughness between the configuration with $r_{t}=0.05$ mm to $r_{t}=0.15$ mm, an increase in the experimental toughness of approximately 1.4 MPa$\sqrt{m}$ is observed. Such a variation is predicted when the critical opening $\Delta_{n}^{cr}$ varies from 3 μm to 5 μm. A smaller increase of $K_{IC}$ is predicted for $\Delta_{n}^{cr}=1$ μm. Thus, a critical opening $\Delta_{n}^{cr}$ ranging from 3 μm to 5 μm results in toughness predictions in agreement with the observations. A smaller values of $\Delta_{n}^{cr}$ does not. These values of $\Delta_{n}^{cr}$ result in a predicted toughness for a sharp crack about $K_{IC}=1.08$ MPa$\sqrt{m}$ to $K_{IC}=1.39$ MPa$\sqrt{m}$, comparable to values available in the literature [26,27,28,29,30,31].

## 4. Discussion and Concluding Remarks

A coupled numerical and experimental methodology to model and characterize the failure of an epoxy thermoset is presented. This is considered for a low cross-link density epoxy of which bulk mechanical response is similar to that of ductile polymers. Thus, its mechanical response is realistically described by the Boyce et al. model (1988) in the absence of failure, but any formulation able to capture softening upon yielding and hardening at continued deformation could be used.

Fracture is described with a rate-dependent cohesive model initially formulated for crazing in amorphous polymers [6,20]. A rate-dependent cohesive model is adopted for the description of the failure process that involved localized plastic deformation with cavitation and void growth. The influence of the cohesive zone kinetics in the toughness variation is observed to be smaller to that in PMMA reported in [7]. In the present case, the kinematics of the crack tip plastic fields govern the failure process and subsequently the nucleation of a crack and onset of propagation. Thus, a simplification of the cohesive model with a rate-dependent formulation can be considered for the loading rates and (room) temperature investigated here, although the failure process is intrinsically rate dependent.

A small-scale yielding, with a boundary layer approach, is used to model mode I fracture. The calculations show that plasticity precedes failure for blunted notches in agreement with the observations. The amount of plasticity almost vanishes when a natural crack is considered, corresponding to an initial notch radius equal to the cohesive critical opening $r_{t}=\Delta_{n}^{cr}$.

The cohesive parameters are identified un-directly from the best fit between the toughness predictions against those measured experimentally. The calibration is validated for configurations with two other notch-tip radii. For the identified cohesive parameters, a prediction of the toughness for a natural crack, with the notch radius equal to the value of the critical opening is derived, with may be difficult to obtain experimentally for such material as machining a natural crack without initial stress and plastic deformation is not easy. The predicted value of $K_{IC}=1.08$ MPa$\sqrt{m}$ for a natural crack is in agreement with data available in the literature [26,27,28,29]. For the configuration with a natural crack, the epoxy under consideration does not exhibit a rate-dependent toughness for the range of loading rates investigated here (${\dot{K}}_{I}$ varying for $1\times 10^{-3}$ MPa$\sqrt{m}$/s to 1 MPa$\sqrt{m}$/s). A different response was found for PMMA (see Saad-Gouider et al. [7]) thus suggesting different failure kinetics for the epoxy compared to the PMMA. This indicated that a single estimate of the fracture toughness for the epoxy provides a measure of its crack resistance. The cohesive critical opening $\Delta_{n}^{cr}$ influences the magnitude of the predicted toughness. A parametric investigation on the influence of the critical opening $\Delta_{n}^{cr}$ on the failure predictions was conducted. It is observed that values ranging from 3 to 5 microns enables capturing the experimental fracture toughness. This range is comparable to the critical opening observed in amorphous polymers at room temperature [22]. This value appears realistic for a low cross-link density epoxy thermoset as considered here but awaits a direct experimental estimate. The present description of the neat epoxy is the first brick towards the analysis of fracture in epoxy-based composites. This will be the purpose of investigated in a forthcoming study.

## Figures and Tables

**Table 1:** parameter set for epoxy resin according to constitutive model described in Section 2.3.

Parameter	Notation	Value
Elastic	$E_{sec}$ (MPa)	1395
	ν	0.38
Viscoplastic	$s_{0}$ (MPa)	140
	*A*(K/MPa)	173
	${\dot{\gamma }}^{0}\;$(s${}^{-1}$)	3.6 $\times \;10^{18}$
Softening	$s_{ss}$ (MPa)	114
	*h* (MPa)	648
Hardening	$C^{R}$ (MPa)	14
	*N*	2.7

**Table 2:** Parameters identified of the cohesive model that describe fracture process of the epoxy resin.

Parameters	Initiation	Thickening			Crack
	$\sigma_{n}^{\mathrm{cr}}$ (MPa)	$\sigma^{c}$ (MPa)	$A^{c}$ (${}^{o}$K/MPa)	${\dot{\Delta }}^{0}$ (mm/s)	$\Delta_{n}^{\mathrm{cr}}$ (μm)
Reference	50	140	173	1× 10${}^{-4}$	3
